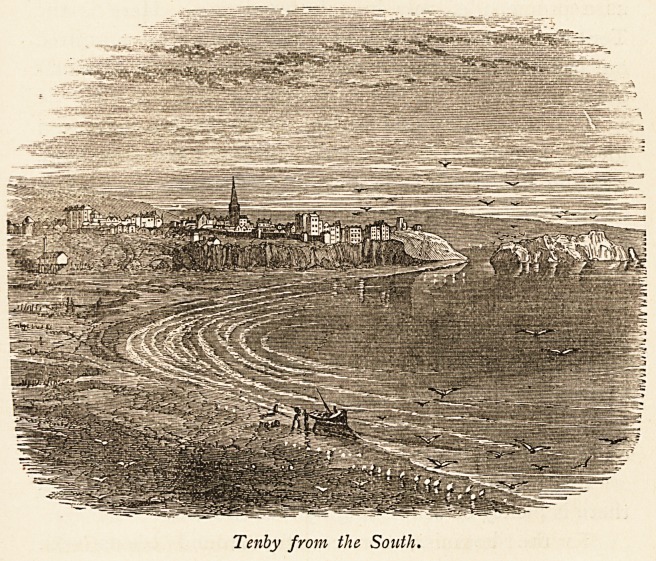# Health Resorts in the West of England and South Wales. VI.—Tenby

**Published:** 1892-06

**Authors:** John Griffith Lock

**Affiliations:** Medical Officer of Health


### Health-Resorts in the West of England and South Wales.

### VI.

### TENBY.

BY

JOHN GRIFFITH LOCK, M.A. Cantab.,

M.R.C.S. Eng., L.R.C.P. Ed., L.S.A.,

*Medical Officer of Health.*[1]

TENBY, which as a health-resort has a summer and a winter phase, is a town with a population of 4,500. It is situated on lime-stone, and runs out as a promontory into the sea with a bay on either side—north and south. It lies in the Bristol Channel, and is protected from the big seas of the Atlantic by Caldy Island on the south.

The North Bay is large, and is bounded by the hills of Carmarthenshire; and on the east by the Worm's Head, a point of Gower Land distant nearly twenty miles. There is very good bathing here. The sands are not so extensive as on the south side, but they are well protected when the equinoctial gales are on. By many persons this bay has been compared to the Bay of Naples. Here is the pier, with its harbour—usually occupied by vessels engaged in trawling, which come from Brixham, Liver-

[1] For the views which illustrate the paper I am indebted to the publisher of *Mason's Guide to Tenby*, Mr. F. B. Mason, Tenby.

pool, Plymouth, and other places, specially in spring and summer, when there is a large quantity of fish in "the Channel."

The South Bay looks out on Caldy Island. Giltar and Penally are on the west. The sands are very extensive and firm, and are much used for walking and riding. Here, too, the greatest amount of bathing is carried on.

*Tenby from the North.*

Within the last few years the Tenby Marine Baths Company has opened new and spacious public baths, adjoining the entrance to the South Sands, where hot and cold (sea and fresh water) baths can be had daily, from 7 a.m. till 8 p.m., and on Sundays for a couple of hours in the morning.

The sands in both bays, besides being used for bathing, are generally frequented by enthusiasts after shells, actiniæ and other zoophytes, of which Mr. Gosse, in his book on Tenby, has given a most interesting account. On the South Sands, near the Castle Hill, which is the extreme eastern point of the town, stands St. Catherine's Rock, insulated at high-water. This island is remarkable for the beautiful caverns with which it is pierced, and as

*Tenby from the South.*

the rocks in places are covered with seaweed, invalids and children can inhale iodine freely.

Promenades are a matter of great importance to invalids and visitors. The chief one is the Castle Hill, which runs out into the sea, and is always, so to speak, "in the sea." Its well-kept walks, grassy slopes, and seats dotted about everywhere, make it a favourite resort. The Esplanade is more stately, and reminds a visitor of the Promenade at Brighton, only in a smaller degree. It is not so private as the Castle Hill, and is overlooked mostly by lodging-houses. During the last few years it has been extended towards the west, and numerous seats have been placed here and there. The Croft is also used as a promenade, only in a lesser degree. Here is the Tenby and County Club, to which visitors are admitted on payment, varying according to the length of membership.

For the beauty and variety of its country walks and lovely scenery, Tenby stands almost unrivalled, considering how much it is surrounded by the sea. Giltar, at the extremity of the South Sands, with its limestone cliffs nearly 200 feet high; Waterwynch, with its interesting little bay; the village of Penally; Gumfreston, with its chalybeate springs; and Saundersfoot, are all within easy walking distances, and are well worthy a visit. For those who cannot walk much, and wish to see the various castles, &c., around Tenby, there is a capital supply of carriages; besides, there are the usual bath-chairs for hire on the sands and about the town.

Boating is very good, and during the summer season there is plenty of sea fishing by line and rod.

To the botanist, the country around teems with treasures, though the inroads of the railway and higher cultivation of the land have done away with many rare plants. Professor Babington told me, on his last visit here, that the Tenby plants numbered about 550, nearly all of which he had observed himself.

Having described how a visitor can occupy himself, and raise the general tone of his health during the summer, let us consider Tenby in the dull months of the year.

The mildness of the climate has for many years been well known to the medical profession and others, but the mania for high altitudes in winter has lessened the number of our winter visitors during the last few years. The climate, though humid, is warm and yet bracing; the difference in day and night temperature is not great. Extreme cold is rare, and snow seldom lies on the ground. There is a great freedom from thunderstorms and fogs by land and sea; and in the depths of winter there is a wonderful purity of sky and a great power of sun. The rainfall is moderate, and it is not so continuous as to compel invalids to stay indoors for twenty-four hours.

The mildness of the climate is proved by the fact that fuchsias, and many other delicate plants, live out of doors all the winter; primroses are usually found in blossom in the hedges in the first week in February; and the goldfish in the pond in my garden have lived through four winters.

Mr. Gower, who has charge of the meteorological instruments here, has sent me the following notes, and I think I had better give them in full:

"For the purpose of correctly determining the climate of any place, it is necessary to take an average of the regular, continuous observations of a number of years. In December, 1890, a meteorological station was established at Tenby, but the data used in this article have been largely taken from the official published records of the observations at St. Ann's, on the South Pembrokeshire coast.

"In common with all south-western counties, the air of Pembrokeshire is humid; the average amount of annual rainfall is not nearly so high, however, as might be inferred. The annual rainfall of Tenby is that of the whole country—35 inches. The rain falls more heavily and more frequently during the night than during the day; the all-day drizzling rains of hilly districts are almost unknown; the geological formation of the peninsula on which Tenby stands is highly favourable to the rapid escape of surface water: thus little discomfort is experienced from rain, and the temperature gains considerably from the latent heat set free by the condensation of the moisture. The high temperature of the water of the ocean doubtless contributes most largely to the exceptional mildness of the climate. At all seasons the readings of the air and sea temperatures approximate each other. The records of the last twenty-five years furnish abundant and conclusive evidence of the equableness of the climate. The limited range of the day and night temperatures, and of the winter and summer means, are very noticeable. The following table shows how the extreme minimum readings compare with those of inland towns. The month of January, 1891, is selected; the temperatures are the lowest observed during the week:

| Week ending | Pembroke. | Hereford. | London. | York. | Cambridge. |
|---|---|---|---|---|---|
| Jan. 10 | 25° | 16° | 15° | 18° | 15° |
| " 17 | 30 | — | 14 | 22 | 7 |
| " 24 | 24 | 10 | 21 | 10 | 11 |
| " 31 | 41 | 34 | 36 | 34 | 33 |

"The month of July, with the extreme maximum readings observed during the weeks given, will show the difference in the readings for the same towns in summer:

| Week ending | Pembroke. | Hereford. | London. | York. | Cambridge. |
|---|---|---|---|---|---|
| July 4 | 61° | 71° | 77° | 72° | 76° |
| " 11 | 59 | 74 | 72 | 72 | 70 |
| " 18 | 68 | 77 | 81 | 78 | 82 |
| " 25 | 61 | 73 | 71 | 70 | 72 |

"Thus, whilst Cambridge had a range of 75 degrees, Pembroke had one of only 44 degrees.

"The temperature of the sea-water is from 50° to 53° in the early spring, and from 60° to 63° in summer.

"There is an almost entire absence of frost and snow, and thunderstorms are of rare occurrence. The day is remarkable for clear blue skies. The average annual amount of sunshine during the decade 1881—1890 was 1,636 hours, or nearly five hours daily. The months of May, June, July, and August averaged seven hours a day. Taking into consideration that only *bright* sunshine is recorded, it will be seen that an extraordinary allowance of what is popularly known as fine weather is apportioned to Tenby.

"Briefly, the climatic advantages possessed by Tenby are: a summer temperature of Southern Norway, a winter temperature of the Mediterranean, an immunity from the inconveniences attending falls of snow and prolonged frosts, much sunshine and fine weather generally, advantages that largely result from a tepid sea that permits of open-air bathing during many months of the year. And though its air is humid, the moisture is rarely condensed into fog. With its equable temperature, and atmosphere pregnant with ozone and iodine, Tenby offers to the invalid in search of a recuperative climate all the advantages and none of the inconveniences of a long sea voyage or a Continental health-resort."

In October, 1887, at Tenby, I had the honour of reading a short paper before a meeting of the South Wales and Monmouthshire Branch of The British Medical Association, on the mortality of Tenby during the fifteen years I had been medical officer of health; and as these statistics gave great interest to those who were present, I think I cannot do better than append them on the opposite page.

I chose twenty-one chief factors of death, and though I have not gone deeply into figures since I drew up this table, I think the average for the last four years would be about the same. For the last two years "The Infectious Diseases (Notification) Act of 1889" has been in force, and I have therefore a greater opportunity of judging of our freedom from contagious and epidemic diseases; and my tables will show how few deaths from these diseases occurred in fifteen years.

It is a well-known fact that, during the cholera epidemic in Bristol many years ago, not one case occurred here, though steamers were regularly plying between the two places, and a great number of persons availed themselves of the opportunity of leaving Bristol.

Many of my readers will be surprised to see how great the mortality has been from consumption. The only reason I can assign is, that most of these deaths occur amongst the poorer class, who, on account of their im-

| | Small Pox | Measles | Scarlet Fever | Typhus | Whooping Cough | Diphtheria | Simple Continued Fever | Enteric Fever | Erysipelas | Pyæmia | Septicæmia | Cancer | Phthisis Pulmonalis | Tubercular Meningitis | Struma | Paralysis | Infantile Convulsions | Heart Disease | Bronchitis | Pneumonia | Old Age |
|---|---|---|---|---|---|---|---|---|---|---|---|---|---|---|---|---|---|---|---|---|---|
| 1872 ... ... ... | 1 | 0 | 3 | 4 | 0 | 0 | 0 | 0 | 0 | 0 | 0 | 3 | 10 | 1 | 1 | 0 | 8 | 5 | 1 | 2 | 12 |
| 1873 ... ... ... | 0 | 0 | 0 | 0 | 0 | 0 | 0 | 0 | 0 | 0 | 1 | 6 | 9 | 1 | 0 | 5 | 5 | 8 | 3 | 0 | 11 |
| 1874 ... ... ... | 0 | 3 | 1 | 2 | 1 | 0 | 0 | 1 | 0 | 2 | 5 | 2 | 4 | 0 | 0 | 1 | 5 | 5 | 7 | 2 | 12 |
| 1875 ... ... ... | 0 | 0 | 0 | 1 | 0 | 0 | 0 | 0 | 0 | 0 | 0 | 2 | 10 | 0 | 1 | 1 | 6 | 6 | 6 | 2 | 6 |
| 1876 ... ... ... | 0 | 0 | 3 | 0 | 0 | 0 | 1 | 0 | 1 | 0 | 0 | 0 | 7 | 0 | 0 | 4 | 2 | 7 | 3 | 6 | 6 |
| 1877 ... ... ... | 0 | 2 | 0 | 1 | 2 | 1 | 0 | 1 | 0 | 0 | 1 | 0 | 3 | 0 | 0 | 8 | 6 | 5 | 8 | 3 | 8 |
| 1878 ... ... ... | 0 | 0 | 0 | 0 | 0 | 1 | 1 | 1 | 0 | 1 | 0 | 0 | 5 | 0 | 0 | 2 | 6 | 7 | 5 | 6 | 13 |
| 1879 ... ... ... | 0 | 0 | 0 | 0 | 0 | 1 | 0 | 0 | 3 | 0 | 2 | 1 | 7 | 1 | 0 | 1 | 8 | 2 | 8 | 6 | 12 |
| 1880 ... ... ... | 0 | 0 | 0 | 0 | 0 | 0 | 2 | 0 | 0 | 0 | 0 | 1 | 8 | 0 | 0 | 1 | 4 | 3 | 6 | 7 | 9 |
| 1881 ... ... ... | 0 | 0 | 0 | 0 | 1 | 0 | 1 | 1 | 1 | 0 | 0 | 2 | 7 | 0 | 0 | 7 | 7 | 5 | 4 | 8 | 3 |
| 1882 ... ... ... | 0 | 0 | 0 | 0 | 0 | 0 | 0 | 1 | 0 | 0 | 0 | 2 | 8 | 0 | 0 | 2 | 2 | 6 | 0 | 7 | 8 |
| 1883 ... ... ... | 0 | 0 | 0 | 0 | 1 | 0 | 0 | 1 | 1 | 0 | 0 | 3 | 9 | 0 | 3 | 1 | 1 | 5 | 7 | 11 | 18 |
| 1884 ... ... ... | 0 | 1 | 0 | 2 | 1 | 0 | 1 | 0 | 0 | 0 | 0 | 4 | 13 | 3 | 1 | 2 | 0 | 4 | 6 | 3 | 9 |
| 1885 ... ... ... | 0 | 0 | 0 | 0 | 0 | 0 | 1 | 1 | 0 | 0 | 0 | 3 | 17 | 1 | 0 | 1 | 3 | 10 | 6 | 0 | 13 |
| 1886 ... ... ... | 0 | 6 | 0 | 0 | 0 | 0 | 0 | 0 | 0 | 0 | 0 | 2 | 7 | 0 | 1 | 2 | 7 | 7 | 9 | 1 | 12 |
| | 1 | 12 | 7 | 10 | 6 | 3 | 7 | 7 | 6 | 3 | 9 | 31 | 124 | 7 | 7 | 38 | 70 | 75 | 79 | 64 | 152 |
| | 71 Contagious and Epidemic. | | | | | | | | | | | 138 Constitutional. | | | | 108 Nervous. | | Circulatory. | 143 Respiratory. | | |

9

provident habits, suffer from poor feeding in the winter. They do well in the summer, but will not provide for the times when they cannot work. In the district round Saundersfoot, three miles from here, there is a mining population, and most of these men suffer from carbonaceous lungs, or black phthisis, and chronic bronchitis. The daughters of these men generally find their way into Tenby as domestic servants; marry here, and often start a weakly and consumptive family. The death of a visitor here from consumption is a rare occurrence.

Renal calculus is a disease almost unknown. Cases which do marvellously well, when sent to Tenby, are those of struma in young persons and children. I have been watching lately a patient with spine disease, who had for three years been quite unable to walk, and who had a large discharging abscess; yet, without any special advantages, the patient can now walk well without the help of a stick. Asthmatical and bronchial patients derive great benefit from a sojourn in this place, and some never have the slightest attack while here.

I hope that the few notes I have put together may not be without interest to some of my readers, who may have no knowledge of my interesting little town, so often said to be—

"LITTLE ENGLAND, BEYOND WALES."

## Figures and Tables

**Figure f1:**
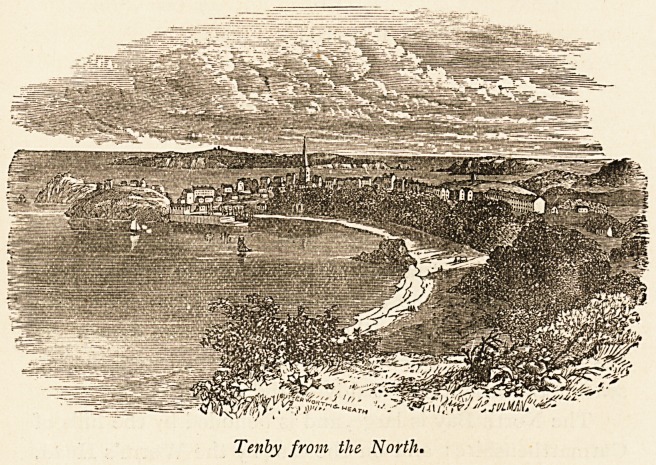


**Figure f2:**